# Postnatal expression profiles of atypical cadherin FAT1 suggest its role in autism

**DOI:** 10.1242/bio.056457

**Published:** 2021-06-08

**Authors:** Jeannine A. Frei, Cheryl J. Brandenburg, Jonathan E. Nestor, Didier M. Hodzic, Celine Plachez, Helen McNeill, Derek M. Dykxhoorn, Michael W. Nestor, Gene J. Blatt, Yu-Chih Lin

**Affiliations:** 1 Program in Neuroscience, Hussman Institute for Autism, Baltimore, MD 21201, USA; 2 Graduate Program in Neuroscience, University of Maryland, School of Medicine, Baltimore, MD 21201, USA; 3 Department of Developmental Biology, Washington University School of Medicine, St Louis, MO 63110, USA; 4 Department of Anatomy and Neurobiology, University of Maryland, School of Medicine, Baltimore, MD 21201, USA; 5 Hussman Institute for Human Genomics and John T. Macdonald Foundation Department of Human Genetics, University of Miami, Miller School of Medicine, Miami, FL 33136, USA

**Keywords:** FAT1, Cadherin, Cerebellum, Granule cells, Autism, Neural precursor cells

## Abstract

Genetic studies have linked *FAT1* (FAT atypical cadherin 1) with autism spectrum disorder (ASD); however, the role that *FAT1* plays in ASD remains unknown. In mice, the function of *Fat1* has been primarily implicated in embryonic nervous system development with less known about its role in postnatal development. We show for the first time that FAT1 protein is expressed in mouse postnatal brains and is enriched in the cerebellum, where it localizes to granule neurons and Golgi cells in the granule layer, as well as inhibitory neurons in the molecular layer. Furthermore, subcellular characterization revealed FAT1 localization in neurites and soma of granule neurons, as well as being present in the synaptic plasma membrane and postsynaptic densities. Interestingly, FAT1 expression was decreased in induced pluripotent stem cell (iPSC)-derived neural precursor cells (NPCs) from individuals with ASD. These findings suggest a novel role for FAT1 in postnatal development and may be particularly important for cerebellum function. As the cerebellum is one of the vulnerable brain regions in ASD, our study warrants further investigation of FAT1 in the disease etiology.

## Abstract (precis)

**Summary:** This study describes a novel role of FAT1 in postnatal brain development and its potential contribution to the etiology of autism spectrum disorder.

## INTRODUCTION

The cadherin superfamily of cell adhesion molecules consists of more than one hundred members that are subdivided into distinct subfamilies, including classical type I and type II cadherins, clustered and non-clustered protocadherins, and atypical FAT cadherins (Hulpiau and van Roy, 2009; Hirano and Takeichi, 2012). Cadherins play widespread roles throughout brain development as they have been implicated in neurogenesis, migration, axon outgrowth, target recognition, synaptogenesis, and synaptic plasticity (Hirano and Takeichi, 2012; Friedman et al., 2015). The vertebrate atypical FAT cadherin family, consisting of four members (FAT1-4) is distinguished from other cadherin subfamilies by their unusually large extracellular domains containing 32 to 34 cadherin repeats (Sadeqzadeh et al., 2014). These extracellular cadherin domains (ECDs) are highly similar among the four FAT cadherins; however, the cytoplasmic domain is less conserved and likely reflects the specific functions of each FAT cadherin (Sadeqzadeh et al., 2014). Depending on the tissue, FAT cadherins act both synergistically and antagonistically to exert their functions (Saburi et al., 2012). Studies in vertebrates have shown that FAT cadherins play critical roles in early development of the nervous system. Rather than conveying molecular codes that specify cell–cell contacts similar to the function of classical cadherins, FAT cadherins are particularly important in the establishment of polarity, including morphogenesis of epithelial structures, neuronal migration, and cellular proliferation (Avilés and Goodrich, 2017).

FAT1 and FAT4 are the most frequently studied members of the family, although all four FAT cadherins have been implicated in cancer development (Sadeqzadeh et al., 2014). Recently, genetic variations in *FAT1* have been associated with neurological disorders, such as bipolar disorder (Blair et al., 2006; Abou Jamra et al., 2008) and autism spectrum disorder (ASD) (Hussman et al., 2011; Cukier et al., 2014; Neale et al., 2012). ASD contains a range of neurodevelopmental conditions categorized by difficulties in social interaction and communication, as well as repetitive behaviors (American Psychiatric Association, 2013). To date, about 1 in 54 children in the United States are diagnosed with ASD (Maenner et al., 2020). Although ASD is characterized by high genetic and phenotypic heterogeneity, many of the genes associated with ASD converge into selective cellular pathways, including those that regulate neural circuit formation (Lin et al., 2016; Hussman et al., 2011; Betancur et al., 2009). Since FAT1 has been associated with ASD, evaluating how this protein affects brain development and regulates circuit formation is important to understand the etiology of this neurodevelopmental disorder.

During embryogenesis, FAT1 is predominantly expressed in epithelial cells and in the central nervous system (CNS) (Ponassi et al., 1999; Dunne et al., 1995). In the developing CNS, FAT1 is expressed in the neuro-epithelium, and later in the proliferating ventricular and subventricular zones of the neocortex (Ciani et al., 2003; Badouel et al., 2015). Knockdown of FAT1 in mice results in increased proliferation of radial precursor cells and disrupted radial migration of neurons in the developing cerebral cortex, indicating a role for FAT1 in corticogenesis (Ciani et al., 2003; Badouel et al., 2015). FAT1 knockout mice are prenatally lethal, probably due to a failure in glomerular slit formation in the kidneys (Ciani et al., 2003). Furthermore, loss of FAT1 leads to severe cranial midline defects and exencephaly, reflecting the important role of FAT1 in early brain development (Cox et al., 2000; Badouel et al., 2015; Ciani et al., 2003).

In contrast to its high expression during embryogenesis, much less is known about the expression and distribution of FAT1 later in development. Depending on the tissue and species investigated, some studies have reported downregulated or absence of FAT1 expression in adult tissue (Ponassi et al., 1999; Dunne et al., 1995), while others have observed low-levels of postnatal expression (Ponassi et al., 1999; Cox et al., 2000). To better understand FAT1 expression beyond embryonic stages, we conducted a detailed analysis of FAT1 protein expression during postnatal brain development in mice at the brain area-, cellular-, and subcellular levels. Furthermore, we investigated the association of FAT1 with ASD by analyzing its expression in neural precursor cells (NPCs) differentiated from induced pluripotent stem cells (iPSCs) derived from individuals with autism compared to those from control individuals. Our findings suggest a potential function of FAT1 in the cerebellum during early postnatal development and strengthen its association with ASD by showing altered expression levels of FAT1 in autism-specific NPCs.

## RESULTS AND DISCUSSION

### The postnatal cerebellum exhibits high levels of FAT1 protein

**Fig. 1.**
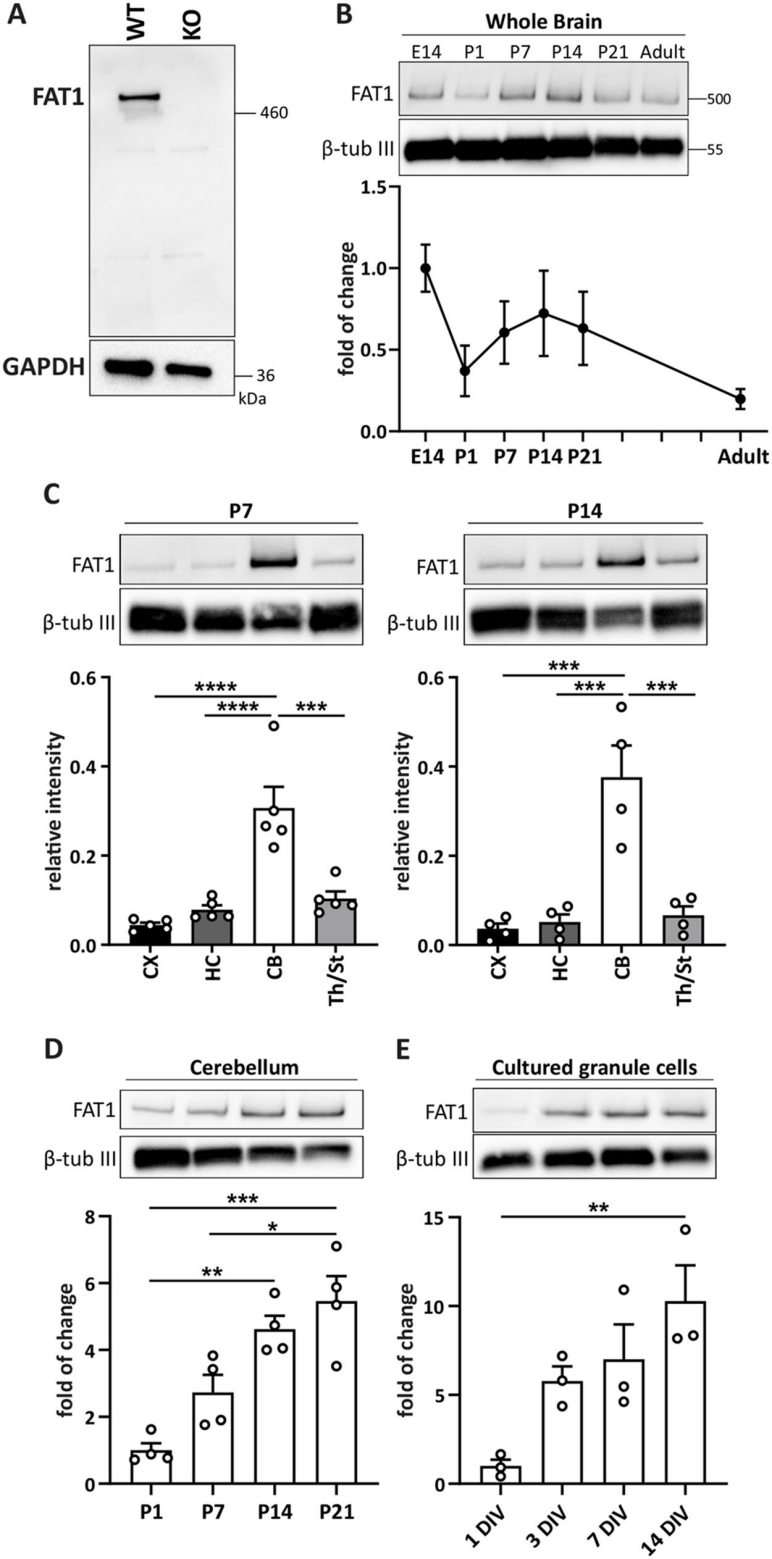
**The postnatal cerebellum exhibits high levels of FAT1 protein.** (A) The specificity of FAT1 antibody was tested by Western blot. Lysates from vascular smooth muscle cells isolated from aortas of wild-type (WT) and FAT1 knockout (KO) mice were probed with anti-FAT1 antibody. The antibody recognizes one band around 500 kDa, the predicted size of FAT1, in the WT but not KO lysate. (B) Temporal expression profile of FAT1 in mouse whole brain collected at E14, P1, P7, P14, P21 and 5-month-old adults. *N*=6 whole brains per age from three independent litters. (C) Spatial expression profile of FAT1 in cortex, hippocampus, cerebellum and thalamus/striatum at P7 and P14 of mouse brain development. P7: ***p=0.0002, ****p<0.0001; P14: ***p=0.0002 CX versus CB, *p*=0.00023 HC versus CB, *p*=0.0005 CB versus Th/St; one-way ANOVA with Tukey's multiple comparison test. *N*=4 samples per brain area with 2–3 pooled brain areas per sample. (D) Temporal expression of FAT1 in the cerebellum at P1, P7, P14 and P21. *p=0.0116, **p=0.0014, ***p=0.0002; one-way ANOVA with Tukey's multiple comparison test. *N*=4 samples per time point with 2–3 pooled cerebellums per sample. (E) Temporal expression profile of FAT1 in cultured cerebellar granule neurons harvested at 1, 3, 7, and 14 DIV. **p=0.0094; one-way ANOVA with Tukey's multiple comparison test. *N*=3 independent cultures.

To thoroughly investigate the distribution of FAT1 in the brain, we analyzed its developmental expression profile in wild-type C57BL/6 mouse brains. The specificity of the anti-FAT1 antibody was first validated by Western blot using whole cell lysates from vascular smooth muscle cells from wild-type and FAT1 knockout mice. The anti-FAT1 antibody recognizes a band above 460 kDa that is not detectable in the FAT1 knockout tissue (Fig. 1A). This antibody was then used to investigate the temporal expression of FAT1 in whole brain tissue of mice at different developmental ages ranging from embryonic day 14 (E14) to adulthood (Fig. 1B). FAT1 was highly expressed at E14, which is in line with previous studies showing elevated *Fat1* mRNA expression in the neuro-epithelium and proliferating germinal zones in the embryonic mouse brain (Ciani et al., 2003; Badouel et al., 2015). At birth, FAT1 protein levels dropped but exhibited a second peak at postnatal day 14 (P14). After P21, FAT1 levels gradually declined and reached lower levels in adulthood compared to E14.

We next analyzed FAT1 expression in different brain areas, including cortex, hippocampus, cerebellum, and thalamus/striatum at P7 and P14 (Fig. 1C). Intriguingly, FAT1 was highly expressed in the cerebellum compared to the other brain areas at both time points. Temporal expression of FAT1 in the cerebellum revealed a significant increase from P1 to P21 (Fig. 1D). We then examined the expression levels of FAT1 in cerebellar granule cells that make up the majority of cells in the cerebellum. Granule cells were dissected from P7 mouse cerebellum and cultured for 1, 3, 7, and 14* *days *in vitro* (DIV). We found a gradual increase of FAT1 expression from 1 DIV to 14 DIV (Fig. 1E), recapitulating the temporal expression profile seen in cerebellum tissues. Together, these findings show that FAT1 is expressed in the postnatal brain, particularly in the cerebellum.

Consistent with previous studies reporting expression of *Fat1* mRNA in adult cerebellum (Ponassi et al., 1999; Rock et al., 2005; Baron et al., 2019), we show that FAT1 protein indeed exhibits relatively high levels in the cerebellum. Our findings from the temporal and spatial expression analysis further indicate that FAT1 may not be required for initial postnatal cerebellar development, such as proliferation and migration of granule cells, but maybe necessary later for the formation of cerebellar neural circuits, including neurite and synapse formation.

### FAT1 is expressed in granule cells and interneurons in the cerebellum and hippocampus

**Fig. 2.**
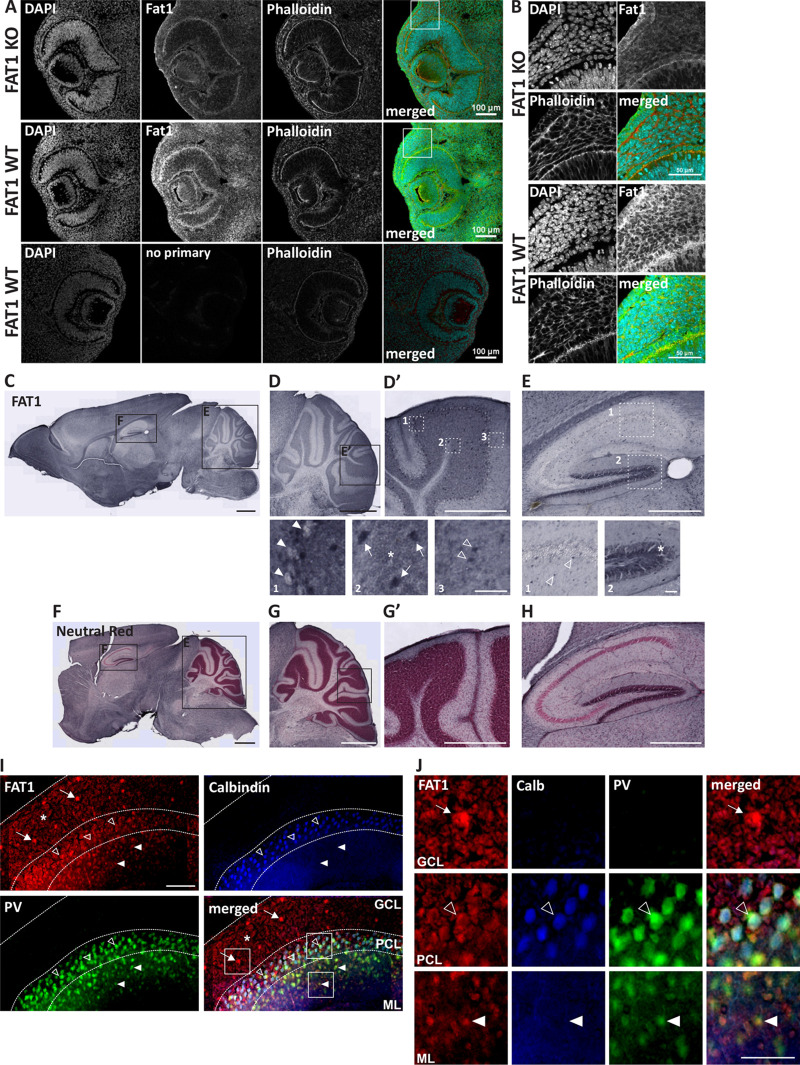
**FAT1 is expressed in granule cells and interneurons in the cerebellum and hippocampus.** (A) The specificity of FAT1 antibody was tested on sagittal sections of embryonic eyes from E13.5 FAT1 KO and WT mice. Scale bars: 100 μm. (B) Magnification of boxed areas depicted in A. FAT1 (green) colocalizes with phalloidin-stained actin (red) in WT but not KO eyes. Sections were counter-stained with DAPI (blue). Scale bars: 50 μm. (C–H) DAB immunostaining of FAT1 and (F–H) FAT1-DAB and neutral red co-staining on P14 mouse brain sagittal sections. Magnifications of cerebellum are depicted in (D), (D′), (G) and (G′) and magnifications of hippocampus are depicted in (E) and (H). FAT1 is expressed in the cerebellum (D,D′) in the surrounding but not in the soma of Purkinje cells (box 1, arrowheads), in the granule cell layer (box 2) in granule cells (asterisk) and Golgi cells (arrows), and in interneurons in the molecular layer (box 3, open arrowheads). FAT1 is also expressed in the hippocampus (E) in interneurons (box 1, open arrowheads) and in granule cells of the dentate gyrus (box 2, asterisk). Note that FAT1 is enriched in cell dense structures of cerebellar granule layer (G,G′) and hippocampal dentate gyrus (H). Scale bars: 1 mm (C,D,F,G), 0.5 mm (E,H), 100 μm (D′,G′), 50 μm (box 1–3 in D′, box 1,2 in E). (I) Sagittal sections of P14 cerebellum co-immunostained for FAT1 (red), calbindin (blue), and parvalbumin (PV; green). Dashed lines indicate layers: GCL, granule cell layer; PCL, Purkinje cell layer; ML, molecular layer. Scale bar: 100 μm. (J) Magnification of GCL, PCL, and ML depicted in merged image in I. Scale bar: 50 μm. FAT1 is expressed in granule cells (asterisk) and Golgi cells (arrows) in the GCL, around calbindin- and PV-positive Purkinje cells (open arrowheads) in the PCL, and PV-positive interneurons (arrowheads) in the ML.

To further investigate which cell types express FAT1, we performed histological examination of FAT1 on sagittal sections of P14 wild-type C57BL/6 mouse brains. FAT1 antibody was first evaluated on FAT1 wild-type and knockout tissues to confirm its specificity (Fig. 2A,B). DAB-staining revealed that FAT1 was expressed by cells throughout the whole brain, including cortex, hippocampus, and cerebellum (Fig. 2C–E). Neutral red staining was performed to highlight the variable cell densities in different areas of the brain (Fig. 2F–H). Interestingly, FAT1 was particularly enriched in the areas of high cell densities, such as the cerebellar granule cells (Fig. 2D,D′) and the dentate gyrus granule cells (Fig. 2E). In addition, FAT1 was detectable in interneurons in both the cerebellum and hippocampus, including Golgi cells in the cerebellar granule cell layer and other interneurons in the molecular layer, as well as in hippocampal interneurons in CA1, CA2, and CA3 regions (Fig. 2D′,E). FAT1 was not detectable in Purkinje cells or pyramidal cells in CA regions (Fig. 2D′,E). We performed immunofluorescence staining to confirm the cell type of cerebellar neurons expressing FAT1 in sagittal sections of P14 mouse brains and co-stained the tissue with calbindin, a marker for Purkinje cells, and parvalbumin (PV), a marker for Purkinje cells and interneurons residing in the molecular layer (Fig. 2I,J). In line with the DAB staining, FAT1 was expressed in granule cells and Golgi cells in the granule cell layer (GCL). Golgi cells contact and modulate the excitatory granule cell-mossy fiber synapses in the cerebellar glomeruli, thus regulating input into the cerebellum (Hashimoto and Hibi, 2012; Hámori and Szentágothai, 1966; Hámori and Somogyi, 1983; Jakab and Hámori, 1988). Furthermore, FAT1 was expressed in PV-positive cells in the molecular layer (ML). Interestingly, FAT1 immunofluorescence was detectable around calbindin- and PV-positive cells located in the Purkinje cell layer (PCL). The presence of FAT1 in PV+-interneurons, e.g. basket and/or stellate cells, as well as around Purkinje cells, indicate that FAT1 may be present in axons of interneurons connecting to the soma of Purkinje cells, such as basket cells. Basket cells inhibit Purkinje cell firing and, as a result, influence cerebellar cortical output to the deep cerebellar nuclei. Based on its increased expression during postnatal cerebellar development, FAT1 has the potential to regulate the development and/or maintenance of various neural circuitries in the cerebellum, including inhibitory synapses on granule cells and Purkinje cells.

### FAT1 localizes to neurites and soma of cultured cerebellar granule cells

**Fig. 3.**
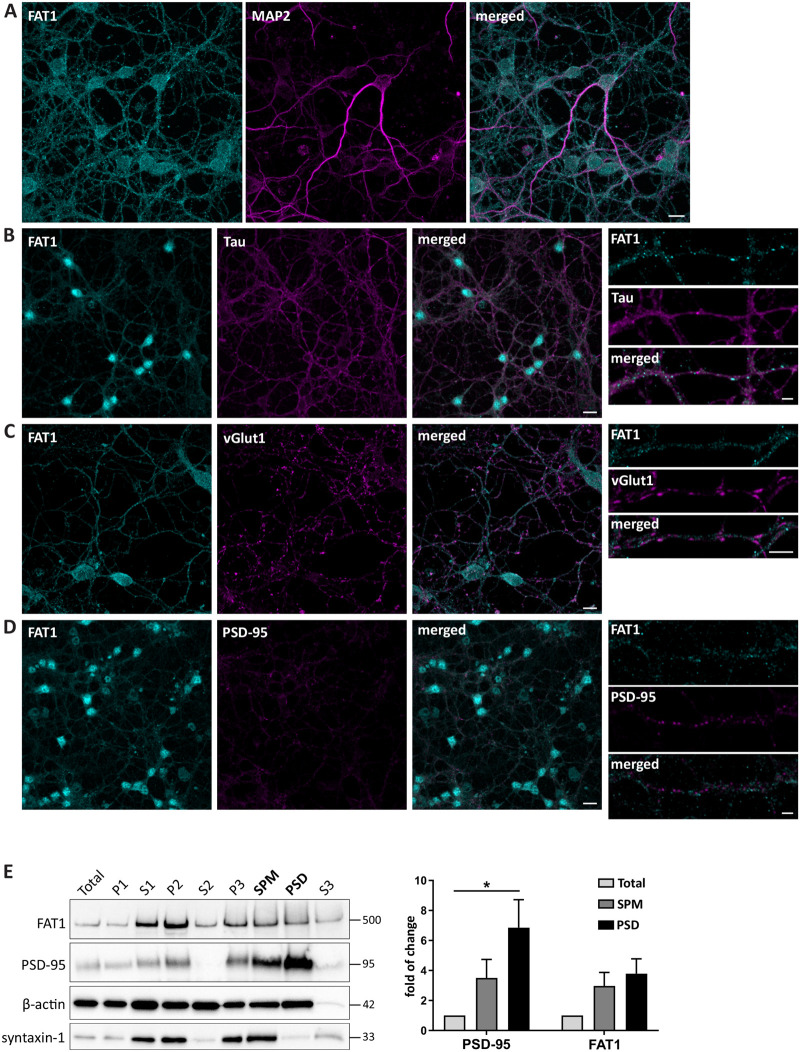
**FAT1 localizes to neurites and soma of cerebellar granule cells.** Confocal images of cerebellar granule cell cultures fixed at 14 DIV and immunostained with FAT1 (cyan) together with different neuronal and synaptic markers (magenta), including (A) MAP2, (B) Tau, (C) vGlut1, and (D) PSD-95. FAT1 is expressed in dendrites, axons, and soma and localizes to vGlut1 and PSD-95 puncta. Scale bars: 10 μm, 2 μm (magnifications in B–D). (E) Synaptic fractionation of P21 cerebellar tissue was performed to determine the subcellular localization of FAT1. Western blots were probed, stripped, and reprobed with markers PSD-95, syntaxin-1 and β-actin to confirm successful separation and purity of different fractions. Total, total protein input; P1, nuclear; S1, cytosol/membranes; P2, crude synaptosome; S2, cytosol/light membranes; P3, synaptosome; SPM, synaptic plasma membrane; PSD, postsynaptic density; S3, synaptic vesicles. *p=0.0422; one-way ANOVA with Tukey's multiple comparison test. *N*=6 mice, two pooled cerebellums per sample.

We next investigated the subcellular localization of FAT1 in cerebellar granule cells by immunocytochemistry. FAT1 colocalized at the MAP2-positive dendrites of cerebellar granule neurons cultured for 14 DIV (Fig. 3A). FAT1 was also present on tau-positive axons (Fig. 3B). In addition, expression was detected in cell bodies of neurons. Synaptic fractionation of the cerebellum was further performed to analyze the enrichment of FAT1 in synaptic compartments (Fig. 3E). We first measured enrichment of PSD-95 expression in synaptic plasma membrane (SPM) and postsynaptic density (PSD) fractions compared to the expression in unfractionated cell lysates. PSD-95 was enriched in SPM by a factor of 3.5±1.2 and in PSD by a factor of 6.9±1.9, which was significant compared to total input. FAT1 was also detected in both the SPM (3.0±0.89) and PSD fractions (3.8±1.0), but was not significantly enriched compared to total input. Consistent with these results, we observed FAT1 puncta associating but not fully colocalizing with the excitatory synaptic markers vGlut1 and PSD-95 in 14 DIV cultured cerebellar granule cells (Fig. 3C,D).

Although FAT1 was not enriched to the same extent as PSD-95, its expression in these fractions implicates possible roles in synapse formation. More evidence for such a role comes from the finding that the intracellular domain of human FAT1 interacts with Homer-1 and Homer-3 scaffolding proteins (Schreiner et al., 2006). These proteins act as adaptors in the postsynaptic density by crosslinking multiple proteins, such as glutamate receptors, and are crucial for postsynaptic function. Both Homer-1 and Homer-3 are expressed in the cerebellum, with distinct localization to the post-synapses of granule cells and Purkinje cells, respectively (Shiraishi et al., 2004). Thus, it is likely that FAT1 interacts with Homer proteins, particularly Homer-1, in cerebellar granule cells to regulate synaptic function.

### FAT1 levels are reduced in iPSC-derived cortical neural progenitors from individuals with ASD

**Fig. 4.**
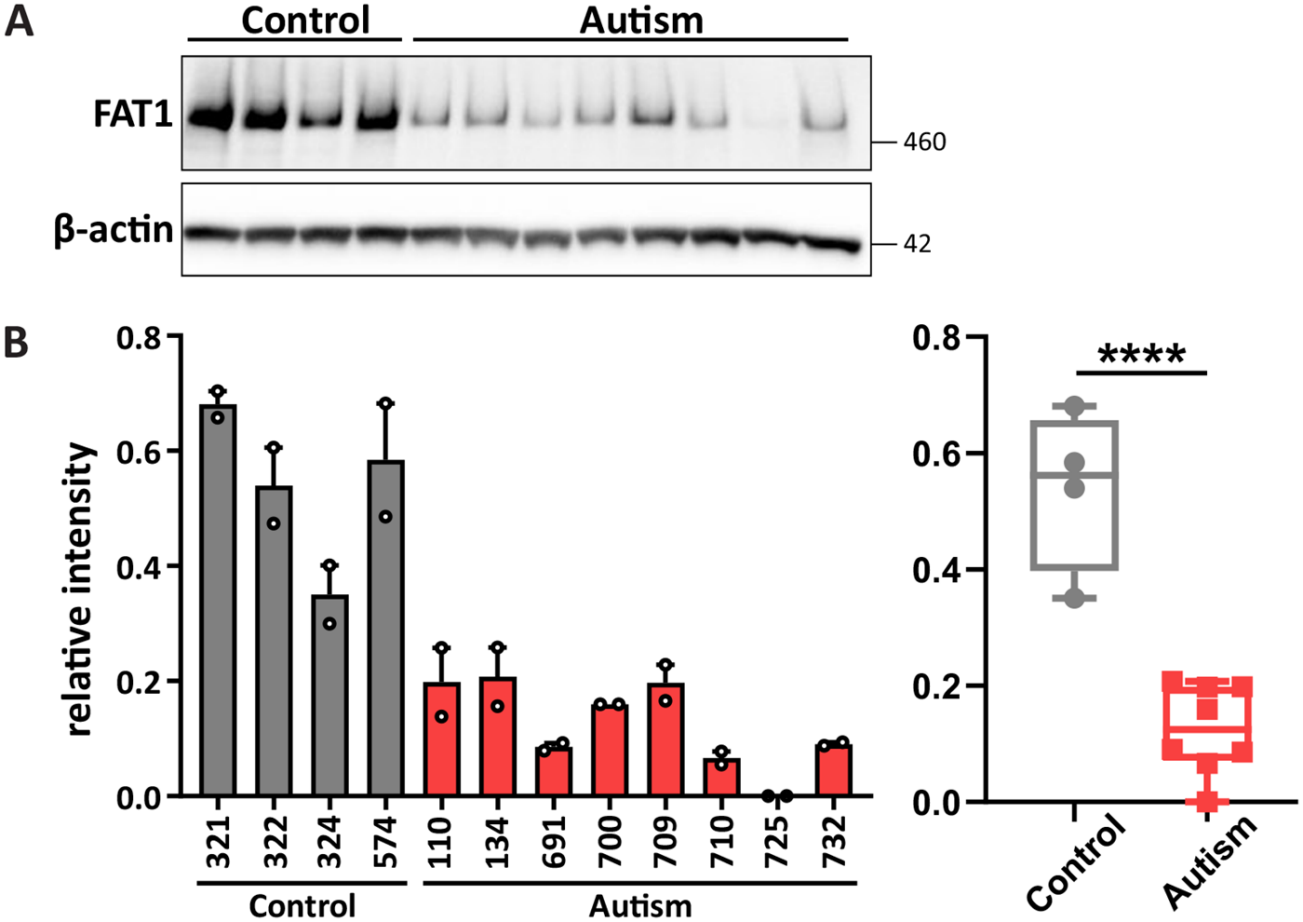
**FAT1 levels are reduced in iPSC-derived cortical neural progenitors from autistic individuals.** (A) Western blot of FAT1 expression in 19 DIV iPSC-derived cortical NPCs from typically-developing control and autistic individuals. (B) Quantification of FAT1 expression represented as a bar graph and as box and whisker plot. ****p<0.0001; unpaired two-tailed *t*-test. *N*=4 control and eight autistic individuals, duplicates of each line.

*FAT1* was identified as an autism-associated gene by a genome-wide association study (Hussman et al., 2011). Further studies performing whole-exome sequencing of ASD families confirmed this association showing *de novo* missense variants (Neale et al., 2012) and inherited damaging missense variants in the *FAT1* gene in autistic family members (Cukier et al., 2014). Here, we examined whether the expression of FAT1 was altered in autism by comparing the expression of FAT1 protein in iPSC-derived cortical NPCs generated from eight individuals with autism and four typically developing control individuals. Some of these lines have been genetically and phenotypically characterized in a previous study, in which none of the lines analyzed carry mutations in the *FAT1* gene (DeRosa et al., 2018). Functional characterization in the same study has also shown reduced migration in autism-specific cortical NPCs at 30 DIV. FAT1 has been implicated in cell migration by intracellular signaling via Ena/VASP proteins to regulate actin assembly and dynamics (Moeller et al., 2004; Tanoue and Takeichi, 2004). We harvested protein lysates from 19 DIV NPCs. Western blot analysis revealed a significant reduction of FAT1 protein levels in the iPSC-derived NPCs from autistic individuals compared to those from the control individuals (Fig. 4). It is plausible that the changes in FAT1 expression observed in the autism-specific NPCs may reflect altered functions of FAT1, such as attenuated migration, during autistic brain development since FAT1 has a well-known function in early development of the nervous system.

The enrichment of FAT1 in the postnatal mouse cerebellum also provides a link of FAT1 to ASD and suggests functions in cerebellar neural circuit formation. The cerebellum has been widely implicated as an affected brain region in autism, with several studies finding decreased numbers of Purkinje cells in postmortem samples from autistic individuals compared to controls (Fatemi et al., 2012; Whitney et al., 2009; Skefos et al., 2014; Bauman and Kemper, 1985; Kemper and Bauman, 1998). In our study, we did not observe FAT1 expression in Purkinje cells. However, FAT1 was detectable around the soma of these cells. Additionally, high FAT1 expression in cerebellar granule cells provides insights for FAT1 playing a critical role in autism. Future studies should address FAT1 expression and function in synapses contacting the cell bodies of Purkinje cells in more detail, as such knowledge could implicate the role of FAT1 in cerebellar development. Examining FAT1 expression in iPSC-derived cerebellar neurons from autistic individuals will also help further elucidate the potential role of FAT1 in autism.

## MATERIALS AND METHODS

### Animals

C57BL/6 mice were obtained from the animal facility of the University of Maryland School of Medicine Program in Comparative Medicine (Baltimore, MD, USA). Mice were housed and cared for by the AAALAC accredited program of the University of Maryland School of Medicine. Female mice were group-housed and male mice were singly housed with *ad libitum* food and water accessibility under a standard 12-h light/dark cycle. Mice of both sexes were used for biochemical analyses and neuronal cell culture preparation. All experiments were performed in accordance with the animal care guidelines of the National Institute of Health and were reviewed and approved by the Institutional Care and Use Committees (IACUC) of the University of Maryland School of Medicine and the Hussman Institute for Autism. *Fat1* KO mice (Gee et al., 2016) were housed at the Mouse Genetics Core facility at Washington University School of Medicine in St Louis and cared for by the Division of Comparative Medicine following animal protocols that strictly adhered to the ethical and sensitive care and use of animals in research. All procedures were approved by the Washington University School of Medicine Animal Studies Committee.

### Antibodies

Antibodies used for biochemical analysis and immunostainings are listed in Table 1.

**Table 1.**
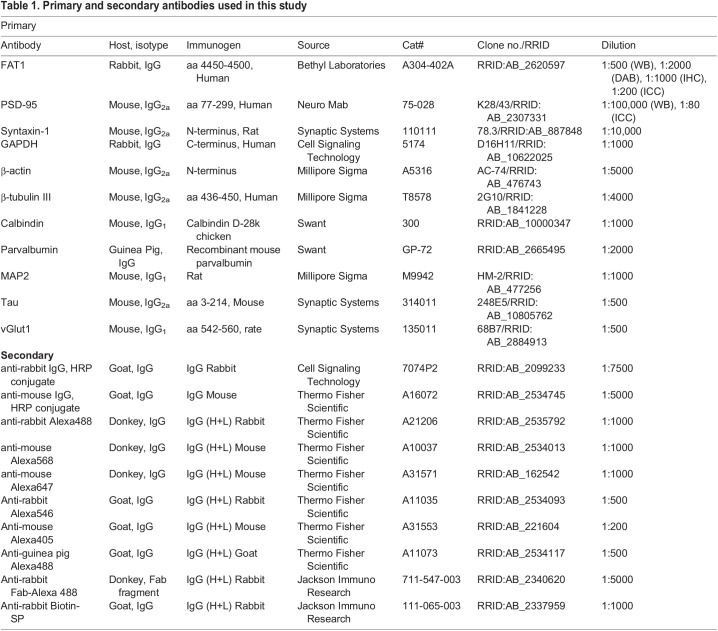
Primary and secondary antibodies used in this study

### Cell culture

Primary cerebellar granule cell cultures were prepared from C57BL/6 mouse cerebellum at postnatal (P) day 7. In brief, brain tissue was dissected and meninges were removed. Tissue was digested in papain and cells were dissociated and plated on tissue culture plates coated with 20 μg/ml poly-D-lysine (Millipore Sigma Cat#P6407). Cerebellar granule cell cultures were maintained in serum-free Neurobasal-A media (Invitrogen Cat#10888022) containing 2 mM L-glutamine (Gibco Cat#25030081), 1% penicillin/streptomycin (Gibco Cat#15140122), and 2% B27 supplement (Gibco Cat#17504044), supplemented with 20 mM KCl. For Western blot analysis, cells were plated at a density of 250,000/cm^2^ and harvested at different time points: 1, 3, 7 and 14 DIV. At the desired DIV, cells were lysed in RIPA buffer (Cell Signaling Technology Cat# 9806S) supplemented with PMSF (Cell Signaling Technology Cat#8553S) and protease/phosphatase inhibitor cocktail (Thermo Fisher Scientific Cat#78442). To determine protein concentration the Pierce BCA protein assay kit (Thermo Fisher Scientific Cat#23227) was used and measurements were performed by the Tecan Spark 10 M multimode microplate reader. For immunofluorescence staining, cerebellar granule cells were cultured on coverslips in 24-well plates at a density of 500,000 cells/well.

### Western blot

Whole brains and different brain areas, including cortex, hippocampus, cerebellum and thalamus/striatum, were collected from both sexes of C57BL/6 mice at different developmental ages: E14, P1, P7, P14, P21 and adult (5-month-old) mice. Mice were euthanized and brains were quickly removed and dissected followed by snap freezing in liquid nitrogen. All tissues were homogenized in RIPA buffer (Cell Signaling Technology Cat# 9806S) supplemented with PMSF (Cell Signaling Technology Cat#8553S) and protease/phosphatase inhibitor cocktail (Thermo Fisher Scientific Cat#78442). FAT1 knockout and wild-type tissue lysates from vascular smooth muscle cells isolated from aortas of *Fat1^flox/flox^* (control) or *Tagln-Cre;Fat1^flox/flox^* (FAT1 knockout) of 5-week-old mice were a gift from Dr Nicholas Sibinga (Albert Einstein College of Medicine, NY, USA). Protein concentrations were determined by Pierce BCA protein assay kit (Thermo Fisher Scientific Cat#23227) and measured by the Tecan Spark 10 M multimode microplate reader. For Western blot analysis, 10 μg of brain or cell lysates were run on 3–8% Tris-acetate SDS-PAGE gels (Nupage Novex Cat#EA0378BOX). Proteins were transferred overnight to 0.22 μm PVDF membranes. Blots were blocked in 5% milk/TBS-T followed by incubation of primary antibodies for 2 h and secondary antibodies for 1 h at room temperature. Blots were imaged using ChemiDoc Touch Imaging System (Bio-Rad) and band intensities were analyzed using the Image Lab software (Bio-Rad). Intensities were normalized to GAPDH, β-actin, or β-tubulin III signal as appropriate.

### Immunohistochemistry

To confirm the specificity of FAT1 antibody, dissected E13.5 FAT1 wild-type and knockout mouse embryos were fixed in 4% paraformaldehyde for 24 h, transferred to 30% sucrose for two days, and embedded in OCT for cryosections at 20 μm on polylysine slides. The primary antibody was diluted in PBS/0.5%Triton-X100/10% donkey serum and incubated overnight at room temperature. Sections were washed in PBS/0.5% Triton-X100 for 1 h and secondary antibody, Phalloidin-Alexa 568 (1:400) and DAPI (1;4000) stains were added in PBS/0.5% Triton-X100/10% donkey serum for 2 h at room temperature. Slides washed for 1 h and mounted (DAKO). Images were acquired on an A1 Nikon confocal microscope. For determining the cell types that express FAT1, P14 C57/BL6 mice were perfused with 4% paraformaldehyde for immunohistochemistry according to standard protocols (Hoffman et al., 2016). Forty micrometer-thick sagittal sections through the cerebellum were cut on a Leica cryostat and sections were stored in glycerol cryoprotectant. Sections were rinsed of cryoprotectant three times for 2 min in a scientific microwave (Ted Pella) at 35°C and 150 watts (all following rinses were performed this way). Sections were blocked with 8% donkey serum in TBS-T for 30 min prior to incubation with primary antibody diluted in 8% donkey serum/TBS-T for 1 h at room temperature followed by incubation for 47 h at 4°C. Sections were rinsed and incubated with secondary antibody for 1 h. After another rinse, A/B solution (1:500; Vector #CatPK-4000) was added for 1 h before incubating sections in DAB solution (95 mM nickel (II) sulfate hexahydrate (Millipore Sigma Cat#N4882), 0.55 mM 3,3′-diaminobenzidine tetrahydrochloride hydrate (Millipore Sigma Cat#32750), 3% (v/v) hydrogen peroxide (Millipore Sigma Cat#216763) in TBS for 20 min. Neutral red staining (0.1%; Millipore Sigma Cat#N7005) was performed for 5 min on selected FAT1-DAB-stained sections. Sections were rinsed, mounted on Superfrost^®^ slides, dehydrated with alcohol, cleared with xylene and mounted with DPX (Millipore Sigma Cat#6522). Fluorescence immunohistochemistry was performed as described above using Alexa Fluor secondary antibodies for 3 h. Imaging was performed with a Zeiss Microbrightfield microscope and Stereoinvestigator software.

### Immunocytochemistry

Cultured granule cell neurons were fixed with 4% paraformaldehyde at 14 DIV for 15 min. Cells were permeabilized with 0.1% Triton-X100 and blocked in 2.5% donkey serum/2.5% BSA for 1 h. Samples were incubated with primary antibodies overnight at 4°C followed by incubation with secondary antibodies for 1 h at room temperature. For PSD-95/FAT1 co-staining, 14 DIV granule cells were fixed in 4% paraformaldehyde/4% sucrose for 10 min. Cells were washed in PBS/0.1 M glycine and permeabilized in 0.3% Triton-X100 in PBS/glycine for 20 min. Samples were blocked in 10% donkey serum/0.2% Triton-X100 in PBS/glycine for 20 min. Cells were incubated with primary antibodies in 5% donkey serum/0.1% Triton-X100 in PBS/glycine overnight at 4°C followed by incubation with secondary antibodies for 1 h at room temperature. Samples were washed in PBS before mounting with Prolong Diamond Antifade mounting medium (Thermo Fisher Scientific Cat#P36934). Images were taken using a Zeiss LSM-780 scanning confocal microscope with a 63× objective/1.40 plan-apochromat oil and 4× zoom.

### Synaptic fractionation

Synaptic fractionation of cerebellar tissue was performed using a protocol for forebrain fractionation published by Bermejo et al. (Bermejo et al., 2014). In brief, cerebellums from P21 mice were quickly dissected on ice followed by homogenization in 0.32 M sucrose in 4 mM HEPES (pH 7.4) at 900 rpm with 12 strokes with a glass-Teflon tissue homogenizer. Low-speed centrifugation at 900× ***g*** for 10 min separated nuclear fraction (P1) from supernatant (S1) that was collected and re-centrifuged at 10,000× ***g*** for 15 min to yield crude synaptosomal fraction (P2) and cytosol/light membranes in the supernatant (S2). Double-distilled H_2_O was added to synaptosomal fraction (P2) for hypo-osmotic lysis followed by centrifugation at 25,000× ***g*** for 20 min to collect pelleted synaptosomes (P3) and vesicular fraction (S3). A discontinuous sucrose gradient was prepared and ultracentrifugation of the gradient at 150,000× ***g*** for 2 h was performed to yield synaptic plasma membrane (SPM) fraction from synaptosomes (P3). The SPM fraction was harvested and re-centrifuged at 200,000× ***g*** for 30 min. Postsynaptic density (PSD) fraction was prepared by incubating SPM in 0.5% Triton-X100 for 15 min and centrifugation at 32,000× ***g*** for 30 min. Western blot was performed to analyze FAT1 expression in different fractions. Intensities of FAT1-positive bands in SPM and PSD fractions were normalized to the total protein input.

### Generation of human iPSC-derived cortical neural precursor cells

Human iPSC lines were derived from peripheral blood mononuclear cells (PBMCs) isolated from whole blood obtained from eight individuals with autism and from four typically developing controls, all from non-Hispanic white (NHW) males (Table 2). All of the ASD patients were recruited through the University of Miami John P. Hussman Institute for Human Genomics. Informed consent was obtained from all participants under a University of Miami Miller School of Medicine Institutional Review Board approved protocol and all experiments were performed in compliance with the guidelines and regulations of the institutional biosafety committee. ASD individuals were ascertained following an ASD diagnosis with following core inclusion criteria: (1) between 3 and 21 years of age, (2) presumptive clinical diagnosis of ASD, (3) an expert clinical determination of an ASD diagnosis was determined using DSM-V (American Psychiatric Association, 2013) criteria supported by the Autism Diagnostic Interview-Revised (ADI-R) (Lord et al., 1994), and (4) intelligence quotient (IQ) equivalent >35 or developmental level >18 months as determined by the Vineland Adaptive Behavior Scale (VABS; Sparrow et al., 2005). The ASD lines 110, 134, 691, 709, 725, 732 had been derived and validated by DeRosa et al., 2018 while lines 700 and 710 have been described in a recent study (Frei et al., 2020 preprint). Control samples (321, 322, 324, 574) were obtained following informed consent under an IRB approved protocol (University of Miami) from cognitively normal individuals that were between 18 and 30 years of age and had no history of ASD or other neurological disorders (e.g. schizophrenia, major depressive disorder). PBMCs were reprogrammed to iPSCs using the CytoTune iPS 2.0 Sendai Reprogramming Kit (Thermo Fisher Scientific Cat#A16517; DeRosa et al., 2012) according to the manufacturer's protocol. The samples were screened for pluripotency and genomic stability according to the published protocol by DeRosa et al. (2018). iPSCs were plated onto mouse embryonic feeders (MEFs) and maintained for 7* *days in mTeSR1 (StemCell Technologies Cat#85850). Selected iPSC colonies showing proliferating cell clusters were selected and dissociated via a 7-min treatment with Accutase in the presence of 20 µM Y27632 (Stemgent Cat#04001210) (Nestor et al., 2015; Phillips et al., 2017). MEF feeders were removed using 0.1% gelatin as previously described (Nestor et al., 2015; Phillips et al., 2017). Neuronal induction was performed as described in DeRosa et al. (2018). In brief, dissociated iPSCs were exposed to Neural Induction Media (NIM) (StemCell Technologies) containing the following small molecules: 10 μM Y27632, 10 μM SB431542 (Stemgent Cat#04001010), 1 μM dorsomorphin (Stemgent Cat#040024), and 1 μM thiazovivin. NPCs were expanded using six-well plates coated with 15 μg/ml Poly-L-Ornithine (Millipore Sigma Cat# P4957) and 10 μg/ml laminin (Invitrogen Cat# 230171015) within an enriched Neurobasal medium consisting of the following: 1:1 mixture of DMEM/F12 (with L-Glutamine; Thermo Fisher Scientific Cat#11320033) and Neurobasal medium (minus phenol red; Thermo Fisher Scientific Cat#12348017), 0.5% N2 supplement (Thermo Fisher Scientific Cat#17502048), 1% B-27 supplement (Thermo Fisher Scientific Cat#12587010), 0.5% non-essential amino acids, 0.5% GlutaMAX (Thermo Fisher Scientific Cat#35050061), 1% Insulin-Transferrin-Selenium-A (Thermo Fisher Scientific Cat#41400045), 1% penicillin/streptomycin (Thermo Fisher Scientific Cat#15140122), 30 ng/ml tri-iodothyronine (Millipore Sigma Cat#T6397), 40 ng/ml thyroxine (Millipore Sigma Cat#T1775), 100 µg/ml bovine-serum albumen (Millipore Sigma Cat#A4161) 60 ng/ml progesterone (Millipore Sigma Cat#P8783), 16 µg/ml putrescine (Millipore Sigma Cat#P7505), 5 μg/ml N-acetyl-L-cysteine (Millipore Sigma Cat#A8199) and 5 μM foreskolin (Millipore Sigma Cat# F6886). At 19 DIV NPCs were lysed in RIPA buffer (Cell Signaling Technologies Cat# 9806S) supplemented with PMSF (Cell Signaling Technologies Cat#8553S) and protease and phosphatase inhibitor cocktail (Thermo Fisher Scientific Cat#78442) and triplicates of each control and autism line were analyzed by Western blot.

**Table 2.**
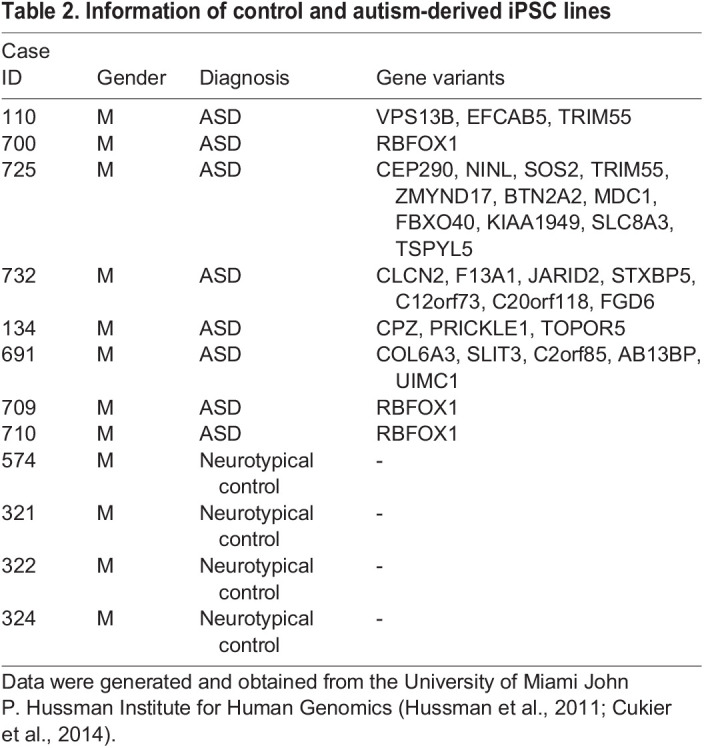
Information of control and autism-derived iPSC lines

### Statistical analysis

Unpaired two-tailed *t*-test was performed to compare two groups and one-way ANOVA with Tukey's multiple comparison test was performed for comparison between three or four groups. *p-*values were considered significant if <0.05. Bar graphs are displayed as mean±standard error of the mean (s.e.m.). All *p-*values and n numbers as well as statistical tests performed are reported in the figure legends. Statistical analysis was performed using Graph Pad Prism 8 software (GraphPad Prism Software, RRID: SCR_002798).
